# Study on dysphagia from 2012 to 2021: A bibliometric analysis *via* CiteSpace

**DOI:** 10.3389/fneur.2022.1015546

**Published:** 2022-12-15

**Authors:** Weiming Sun, Xizhen Kang, Na Zhao, Xiangli Dong, Shilin Li, Gaoning Zhang, Guanxiu Liu, Yang Yang, Chafeng Zheng, Guohua Yu, Lang Shuai, Zhen Feng

**Affiliations:** ^1^Department of Rehabilitation Medicine, The First Affiliated Hospital of Nanchang University, Nanchang, China; ^2^Jiangxi Medical College, Nanchang University, Nanchang, China; ^3^Department of Rehabilitation Medicine, Jiangxi Provincial People's Hospital, Nanchang, China; ^4^Department of Psychosomatic Medicine, The Second Affiliated Hospital of Nanchang University, Nanchang, China; ^5^Department of Rehabilitation Medicine, Zhujiang Hospital of Southern Medical University, Guangzhou, China; ^6^School of Life Science, Nanchang University, Nanchang, China

**Keywords:** swallowing disorder, scientometric review, visualization, research trend, Web of Science

## Abstract

**Objectives:**

This study aims to review the documents on dysphagia, summarize the research direction, analyze the research hot spots and frontiers, report the research trends, and provide new ideas for future development in the field *via* CiteSpace.

**Methods:**

We retrieved articles on dysphagia published between 2012 and 2021 from the Web of Science Core Collection database. We downloaded the entire data and utilized CiteSpace version 5.8.R3 (64-bit) to analyze the number of publications annually, cited journals, countries, institutions, authors, cited authors, cited references, and keywords. We visualized the data with a knowledge map, collaborative network analysis, cluster analysis, and strongest citation burst analysis.

**Results:**

We obtained 14,007 papers with a continually increasing trend over time. The most productive country and institute in this field were the United States (4,308) and Northwestern University (236), respectively. Dysphagia (5,062) and Laryngoscope (2,812) were the most productive journals, Elizabeth Ward had the highest number of publications (84), and Logeman et al.'s article (centrality: 0.02) was the most referenced. The most common keywords were dysphagia, management, quality of life, deglutition disorder, diagnosis, aspiration, prevalence, children, outcome, and oropharyngeal dysphagia.

**Conclusion:**

This study analyzed the current literature on dysphagia *via* CiteSpace and identified its research hot spots and frontiers. The prevalent global trends in dysphagia research and the growing public awareness about healthcare and quality of life suggest that research on dysphagia will gain popularity with further breakthroughs.

## Introduction

The literal definition of the term dysphagia is “eating disorders.” However, clinical practice describes it as the obstruction and stagnation of the pharynx, sternum, or esophagus caused by the obstruction of food transportation from mouth to stomach and cardia (1). If this obstruction is accompanied by pain, it is regarded as dysphagia, and if it is associated with persistent obstruction and bolus retention, it is classified as food incarceration (2). Dysphagia may result from neuromuscular disease or mechanical obstruction (3). It can be classified into two types, namely, oropharyngeal dysphagia caused by pharyngeal and upper esophageal sphincter dysfunction, and esophageal dysfunction caused by a malfunctioning esophagus (4). The onset of dysphagia is marked by difficulties in swallowing, coughing, choking, or aspiration (5). Its progression can further cause serious complications, such as malnutrition, dehydration, aspiration pneumonia, and respiratory failure, and can even increase the fatality risk (6). Dysphagia is a widely prevalent disorder in society, with a significant influence on individuals, families, and paramedics (7). Studies (8–10) suggest that dysphagia adversely affects a patient's quality of life. Psychological research highlights that swallowing disorders impart psychological burdens to patients causing anxiety and depression and increasing their visceral sensitivity (11). The increased physical, social, and psychological needs of patients with dysphagia further burden the caregivers (12).

Dysphagia may occur at varying ages (13) and is closely linked with an increased risk of death or dependency, the occurrence of pneumonia, poor quality of life, and prolonged hospital stay (14). Studies report that approximately 8.1–80% of stroke patients, 11–81% of patients with Parkinson's disease, 27–30% of patients with traumatic brain injury, and 91.7% of patients with community-acquired pneumonia (15) experience difficulty in swallowing. However, these risks and symptoms are typically not the patient's primary complaint and are thus overlooked, posing a significant hidden risk to them. Diagnosis and treatment of dysphagia are continuously emerging (16, 17). An assessment of the accuracy and reliability of a dysphagia screening tool and its correct and rational use may help identify dysphagia and avoid complications (18). However, several issues may persist (19).

We utilized bibliometrics by searching relevant journals, articles, authors, and topics, which allowed us to track the prevailing trends and distinguish the gaps (20). It can help gauge the scholarly impact of a scientific publication (21). CiteSpace is a modeling software for bibliometrics, which supports various types of bibliometric research, including institutional citation analysis, author collaboration network analysis, and visualization of topic and field co-occurrence. It uses scientific mapping programs to carry out visual analysis and research on the structure, dynamic models, and trends in the desired field (21). This allows researchers to intuitively identify the evolutionary path of the discipline frontier and classic basic documents.

The metrological analysis of dysphagia is also trending. However, CiteSpace analyses on and around dysphagia are few. In this study, we performed a bibliometric analysis of publications of related references derived from the Web of Science (WOS) database from 2012 to 2022 by utilizing the CiteSpace software. Subsequently, we have provided an overview of the achievements, future research trends, and hot spots in this research domain.

## Methods

### Data acquisition

Web of Science is an information service platform developed by Clarivate Analytics that supports the retrieval of literature about natural sciences, social sciences, arts, and humanities, with data from journals, books, patents, conference proceedings, and online resources (e.g., free and open resources) (22). It is a generally acceptable database that can be used by scholars globally. The publications indexed in the WOS represent a core part of the research process (23). Therefore, we chose the results from the Web of Science Core Collection (WoSCC) database.

**Figure 1:**
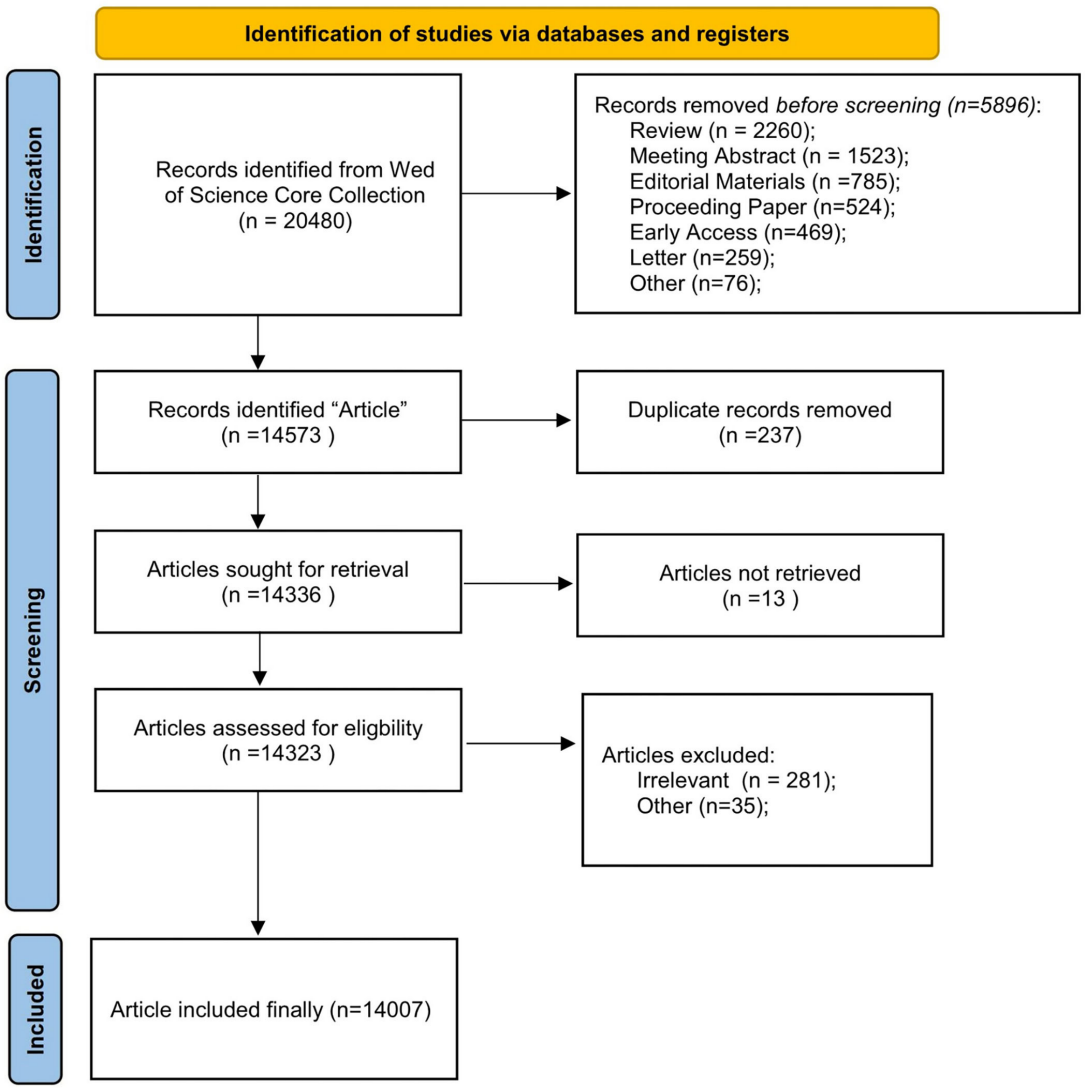
The flowchart of included and excluded studies.

All data were obtained from WoSCC on 4 July 2022. We adopted the following data retrieval strategy: (i) Topic = dysphagia or swallowing disorder or swallowing difficulty or swallowing impairment or aphagia or deglutition difficulty or swallowing dysfunction or ingurgitation difficulty or swallowing problem; (ii) Document Type = article; and (iii) IC Timespan = 2012–2021. We retrieved 14,573 “articles” on WoSCC; of which, 14,007 “articles” were included finally after removing duplicate and irrelevant “articles.” Full records and cited references were selected in plain text format and downloaded for further analysis. The flowchart is shown in Figure 1.

### Analysis tool

CiteSpace is one of the bibliometric analysis software invented by Dr. Chaomei Chen (24–26). CiteSpace, also known as citation space, is a citation visual analysis software that focuses on analyzing the knowledge contained in scientific literature and gradually develops under the background of scientometrics and data visualization (27). This study utilized CiteSpace V.5.8.R3 (64-bit) to analyze the existing studies correlated to dysphagia, aiming to provide scientific and intuitive support for clinicians and researchers in this field. This would help understand the current state and trends in the field and provide new ideas for future development.

We used the software to identify the citation bursts for the research hot spot, keyword, author, research institution, journal, country, and publication year. A visual knowledge graph consists of nodes and links. The different nodes in the CiteSpace map represent elements like institutions, authors, countries, and references that are cited, and the links between the nodes on behalf of collaborative/co-occurring or co-cited relationships. The size of nodes represents the frequency or amount. Different colors refer to different years; darker and light colors indicate previous and recent years, respectively. Purple circles indicate centrality. Nodes with high centrality are often considered turning points or pivot points in the field (28, 29).

## Results

### Annual publication years

**Figure 2:**
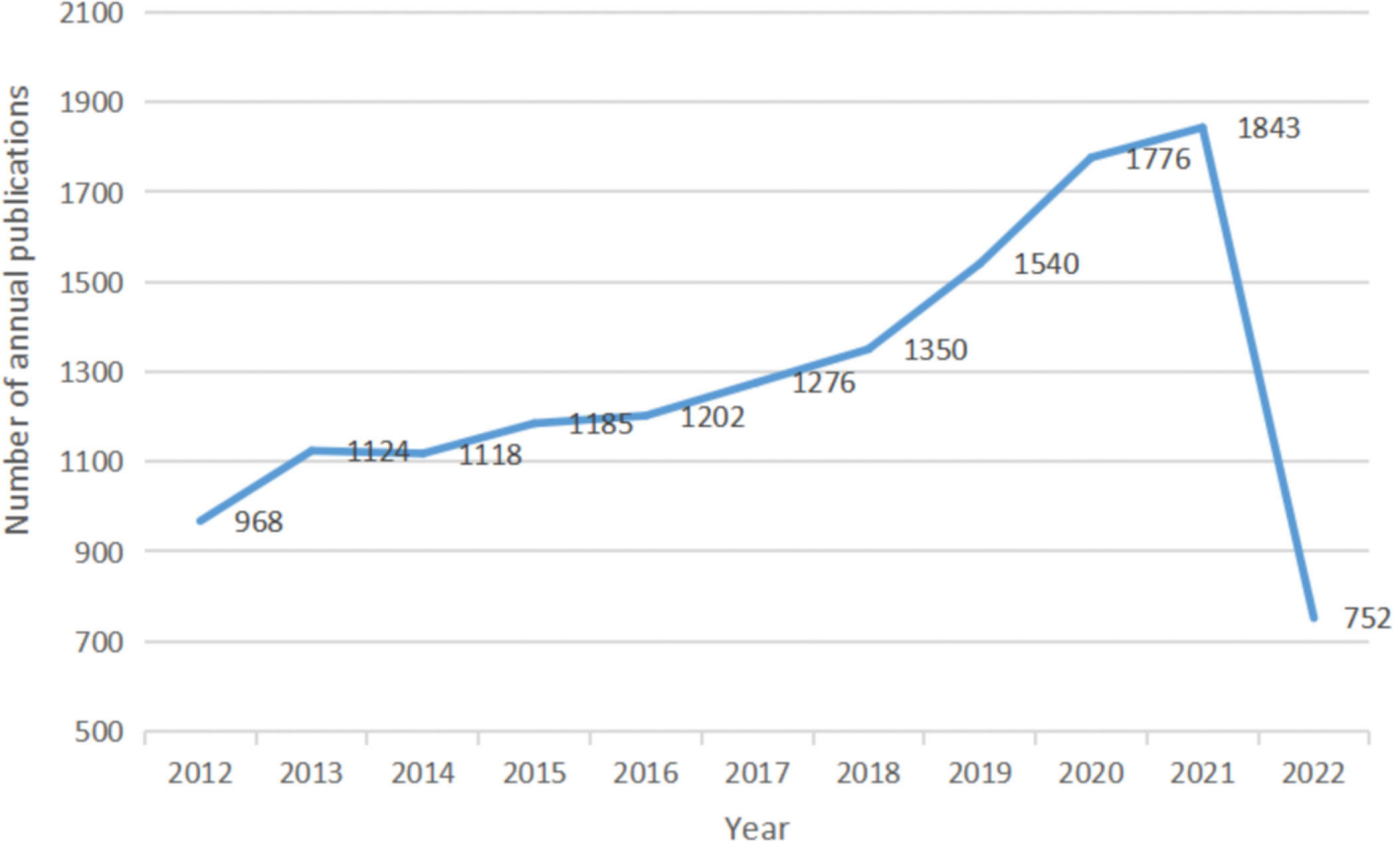
The number of articles related to dysphagia published annually from 2012 to 2022.

As shown in Figure 1, the number of research articles on the subject is significantly expanding, and the number of articles published increased from 1,030 in 2012 to 1,912 in 2021. In addition, a sharp growth period was observed from 2018 to 2021, while a steady growth period was observed from 2012 to 2018. We look forward to the publication of more articles given that 2022 is ongoing, as shown in Figure 2.

### Analysis of country

**Figure 3:**
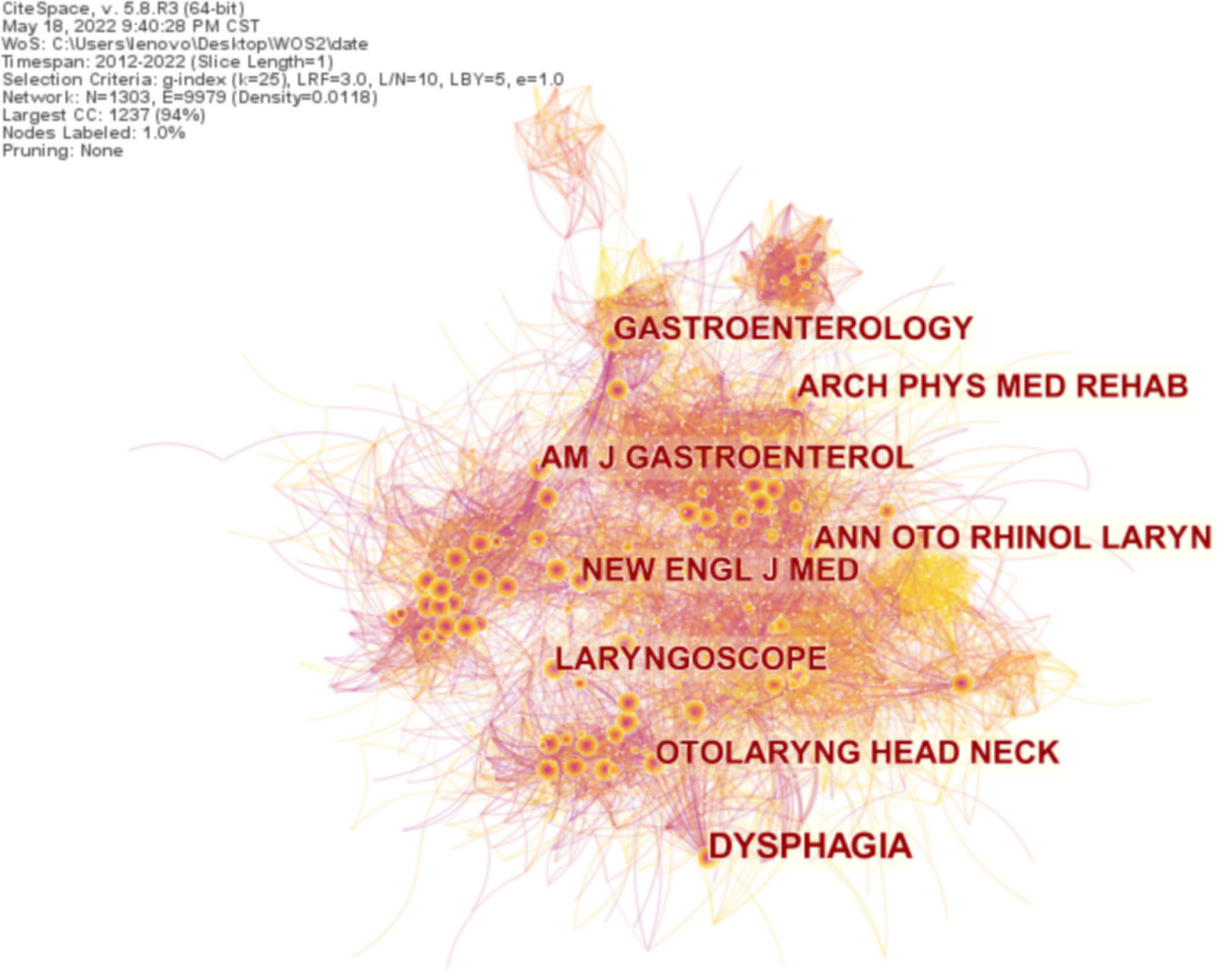
Map of countries distribution and cooperative relations on dysphagia from 2012 to 2022. The larger the circle, the more often it is quoted. The shorter the distance between the two circles, the more the cooperation between the two journals.

To explore the relationship between articles published in each country, we analyzed all articles on dysphagia from 2012 to 2022 in 1-year slices. It generated a country-specific distribution map, which is shown in Figure 3, with a merged network of 155 nodes and 1,090 links. Nodes and the connections between them reveal countries and partnerships, respectively. The size of the circle represents the number of publications in a country; the shorter the distance between the two circles, the more the cooperation between the countries. The orange ring outside the circle represents centrality.

The top ten productive countries in this area of study are shown in Table 1; the US tops the list with 4,038 publications, followed by Japan with 1,508 publications, and then China with 1,227 publications. In addition, the number of documents issued by the US far exceeds those issued by other countries. Therefore, the centrality is higher than that of other countries, indicating its increased influence and cooperation.

**Table 1:** The top ten productive countries and centrality in the research field of dysphagia from 2012 to 2022.

**Ranking**	**Country**	**Frequency**	**Centrality**
1	United States	4,308	0.21
2	Japan	1,508	0.04
3	China	1,227	0.01
4	England	867	0.15
5	Germany	843	0.09
6	Italy	787	0.07
7	Australia	676	0.06
8	South Korea	665	0.00
9	Canada	546	0.02
10	France	502	0.14

### Analysis of the institute

**Figure 4:**
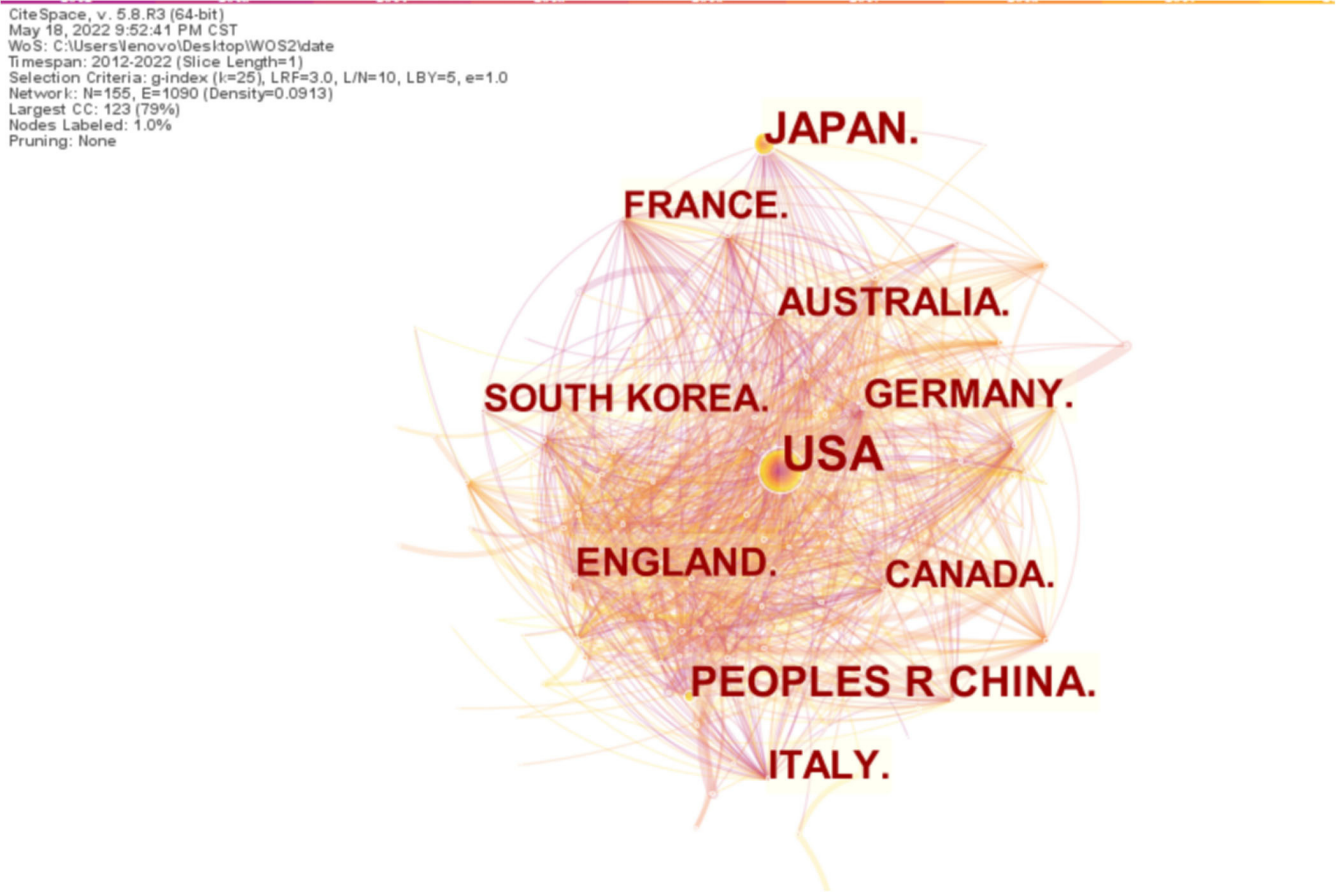
Map of institutions distribution and cooperative relations on dysphagia from 2012 to 2022. The larger the circle, the more articles are published in that country. The shorter the distance between the two circles, the more the cooperation between the countries.

Setting institution as the node type can generate a distribution map of institutions with a merged network of 547 nodes and 3,927 links, as shown in Figure 4. The top ten institutions in this field of study are depicted in Table 2. Northwestern University leads the research on swallowing disorders, with 236 published articles and a centrality of 0.05. It is followed by Mayo Clinic, with 212 published documents and a centrality of 0.08. The University of Queensland, which ranked third in productivity, published 189 articles, with a centrality of 0.04. Table 2 highlights that the top ten institutions are universities whose influence cannot be ignored.

**Table 2:** The top ten productive institutions and centrality in the research field of dysphagia from 2012 to 2022.

**Ranking**	**Institution**	**Frequency**	**Centrality**
1	Northwestern University	236	0.05
2	Mayo clinic	212	0.08
3	University of Queensland	189	0.04
4	Johns Hopkins University	186	0.11
5	University of Toronto	167	0.10
6	University of Florida	142	0.03
7	University of Pittsburgh	136	0.04
8	University of Wisconsin	132	0.01
9	University of Milan	125	0.03
10	Seoul National University	119	0.03

### Analysis of authors

**Figure 5:**
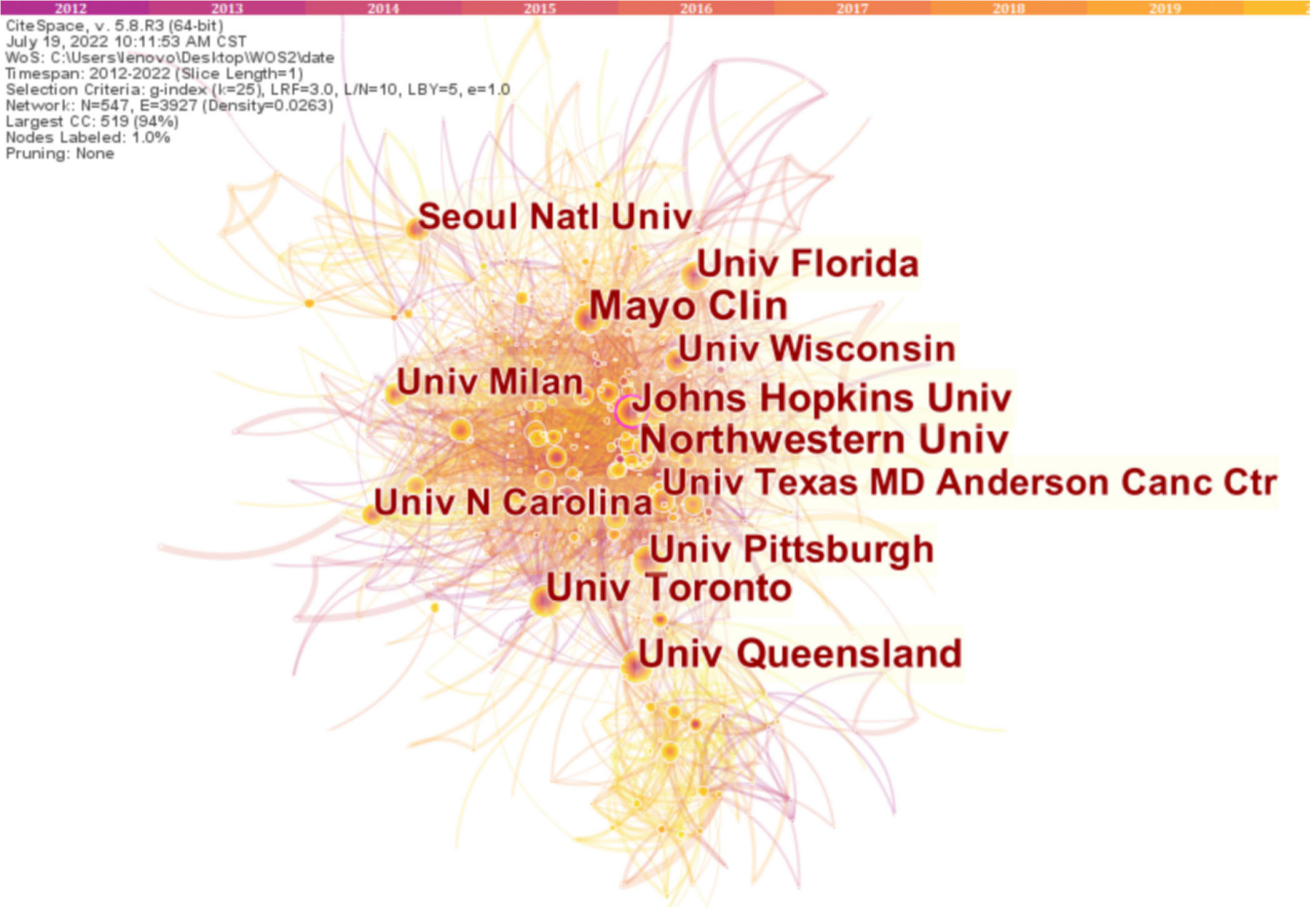
Map of authors distribution and cooperative relations on dysphagia from 2012 to 2022. The larger the circle, the more articles are published in that institution. The shorter the distance between the two circles, the more the cooperation between the institutions.

Articles published between 2012 and 2022 were chosen with a time slice of 1 year. The node type chose an author for the analysis *via* CiteSpace and then gained the co-authorship network map with a merged network of 688 nodes and 1,603 links, as shown in Figure 5. The size of the circle represents the number of articles published by the author; the shorter the distance between the two circles, the more the Connection between the authors. Different colored nodes signify different meanings: the purple node represents early published articles, while the orange node represents recently published articles.

The top ten productive authors in this area of study are shown in Table 3. In terms of performance activity, Elizabeth Ward with a published frequency of 84 ranked first, followed by Catriona Steele and Haruka Tohara. This kind of analysis can provide other researchers with highly personalized scientific information (30).

**Table 3:** The top ten productive authors and centrality in the research field of dysphagia from 2012 to 2022.

**Ranking**	**Author**	**Frequency**	**Centrality**
1	Elizabeth Ward	84	0.11
2	Catriona Steele	60	0.08
3	Haruka Tohara	53	0.09
4	Katherine Hutcheson	50	0.05
5	Ervin Sejdic	44	0.01
6	Evan Dellon	43	0.04
7	John Psndolfino	42	0.07
8	Rainer Dziewas	40	0.03
9	Hidetaka Wakabayashi	39	0.04
10	Peter Belaysky	36	0.02

### Analysis of co-cited authors

**Figure 6:**
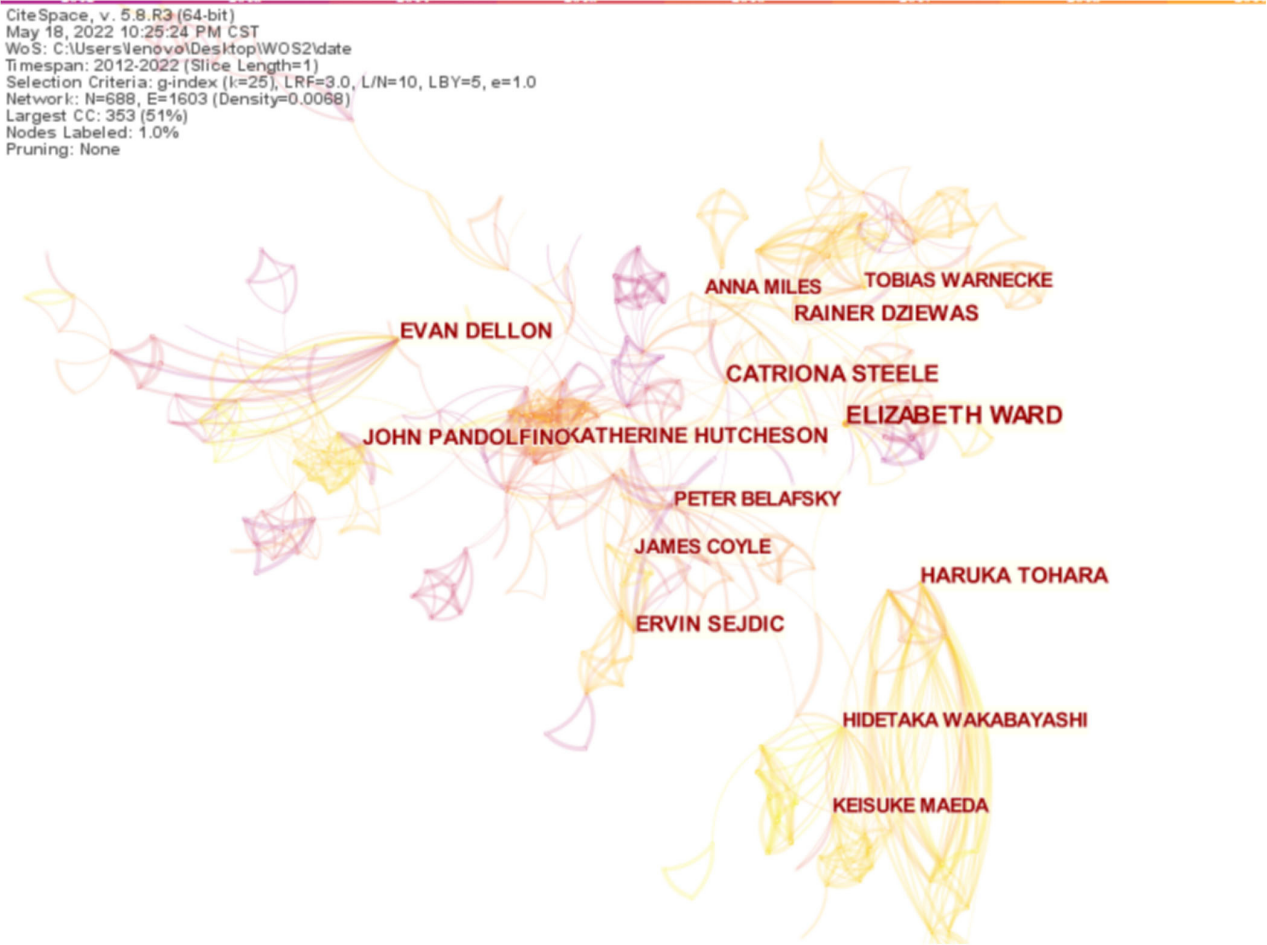
Map of cited author distribution and cooperative relations on dysphagia from 2012 to 2022. The size of the circle refers to the number of articles published by the author, and the shorter the distance between the two circles, the more the connection between the authors.

CieSpace was used to generate a cited author map comprising 1,125 nodes and 8,108 links, as shown in Figure 6. Unfortunately, the top cited author with 1,285 publications is anonymous, followed by Logeman JA with 996 published documents and a centrality of 0.02, followed by Rosenbek JC with 944 published documents and a centrality of 0.05. The top ten cited authors researching on swallowing disorders are shown in Table 4.

**Table 4:** The top ten cited authors and centrality in the research field of dysphagia from 2012 to 2022.

**Ranking**	**Cited author**	**Frequency**	**Centrality**
1	Anonymous	1285	0.00
2	Logemann JA	996	0.02
3	Rosenbek JC	944	0.05
4	Kahrilas PJ	741	0.10
5	Crary MA	720	0.03
6	Martino R	694	0.03
7	Steele CM	687	0.02
8	Robbins J	609	0.02
9	Langmore SE	578	0.03
10	Belaysky PC	527	0.08

### Analysis of co-cited journals

Co-cited journals are those that have been cited by other scholars jointly and represent the journal's impact and contribution to the relevant field. Through the analysis of the cited journal, we can obtain the distribution of the key sources of knowledge in a particular field to find effective information efficiently. The number and influence of publications in the research field are highly correlated with the active degree and attention of research in that field, which represents the future research trends and direction of the industry.

**Figure 7:**
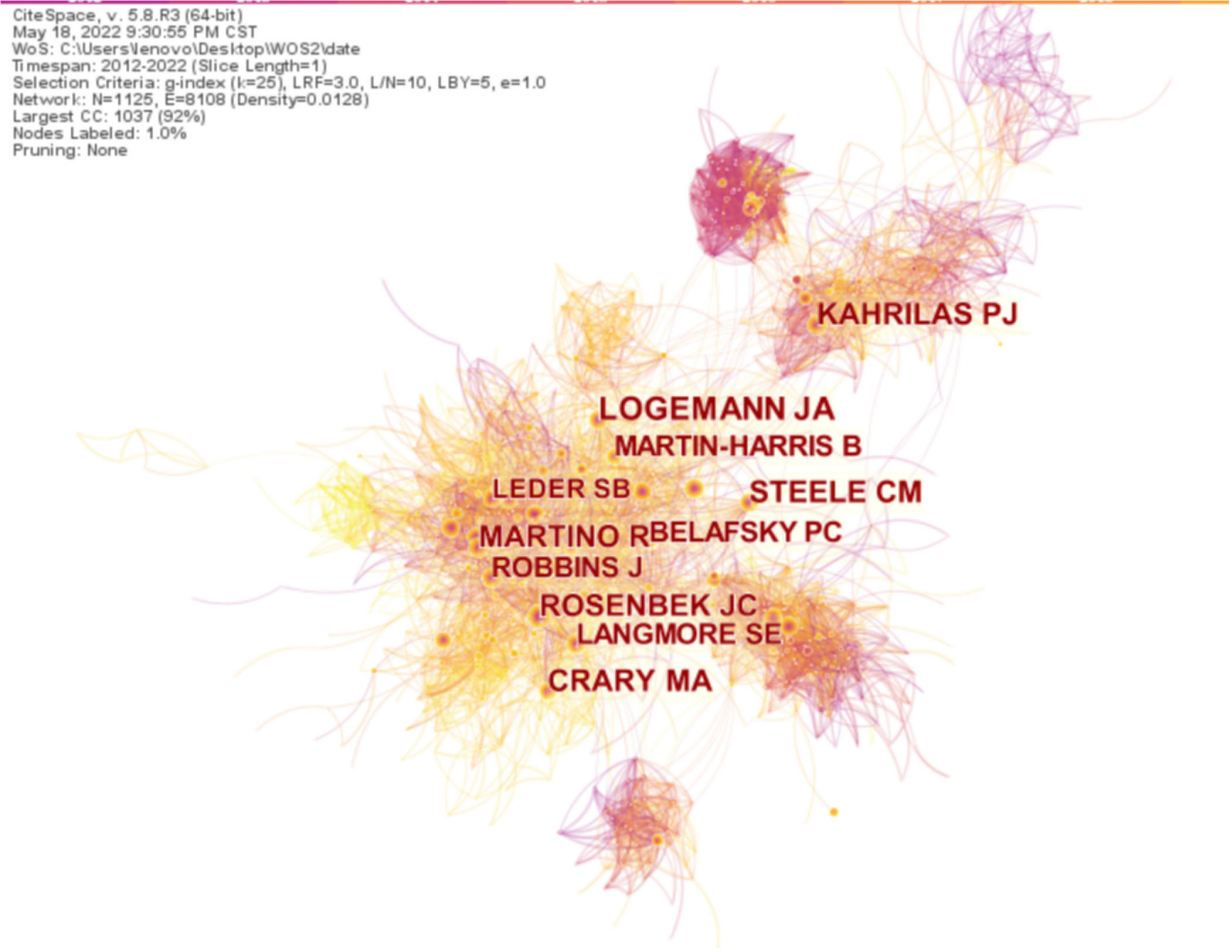
Map of cited journals distribution and relations on dysphagia from 2012 to 2022. The shorter the distance between the two circles, the more the cooperation between the two cited authors.

All articles on dysphagia from 2012 to 2022 were analyzed with 1-year slices and node type of cited journal, generating a distribution map of cited journals with a merged network of 1,312 nodes and 9,982 links, as shown in Figure 7. Nodes and the connections reveal cited journal and their relationships, respectively. The size of the circle represents the frequency of the cited journal; the bigger the circle, the more often it is quoted, and the shorter the distance between the two circles, the more cooperation between the two journals. The orange ring outside the circle represents centrality.

The top ten cited journals in this area of study are shown in Table 5. It suggests that the most frequently cited journal is Dysphagia. Furthermore, highly influential magazines, for example, the *New England Journal of Medicine* and *The Lancet*, are on the list. The vast majority of cited journals are from the United States.

**Table 5:** The top ten cited journals and its influencing factors (IFs) in the research field of dysphagia from 2012 to 2022.

**Ranking**	**Cited journal**	**Frequency**	**IF (2022)**	**Country**
1	Dysphagia	5,062	2.733	United States
2	Laryngoscope	2,812	2.970	United States
3	Gastroenterology	2,329	33.883	United States
4	New England journal of medicine	2,286	176.079	United States
5	Annals of otology rhinology and laryngology	2,101	1.973	United States
6	American journal of gastroenterology	2,061	12.045	United States
7	Archives of physical medicine and rehabilitation	1,971	4.060	United States
8	Otolaryngology-head and neck surgery	1,906	5.591	United States
9	Neurology	1,832	11.800	United States
10	Lancet	1,752	202.731	England

### Analysis of co-cited references

**Figure 8:**
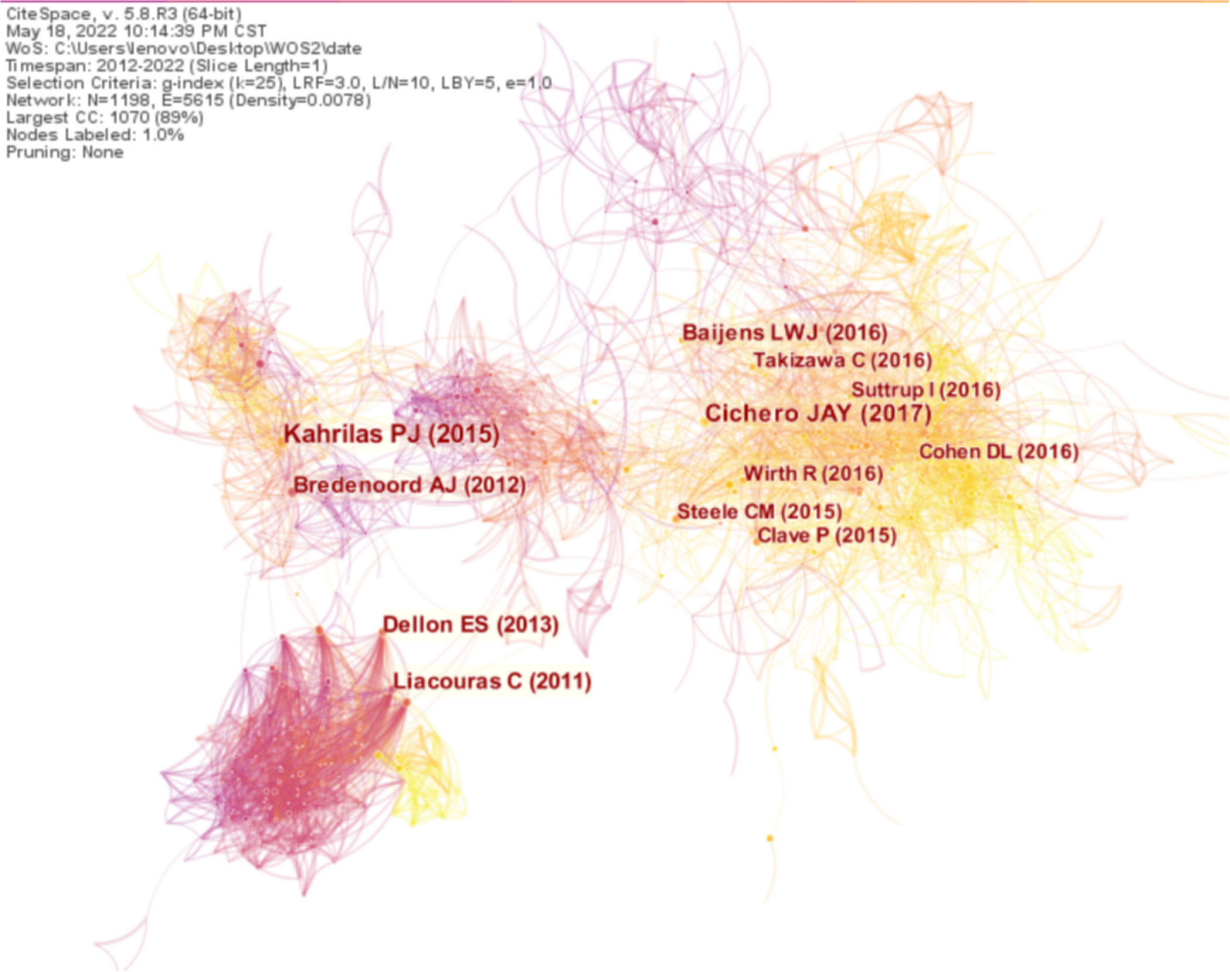
Map of cited references distribution and cooperative relations on dysphagia from 2012 to 2022. The shorter the distance between the two circles, the more the cooperation between the two cited references.

Selecting reference as node type, setting the year of publication span of the article between 2012 and 2022, and choosing the time slice as 1 year generate a co-citation network map with a merged network of 1,198 nodes and 5,615 links, as shown in Figure 8. The size of the circle represents the frequency of cited references; the larger the circle, the more times the article is cited. Furthermore, the shorter the distance between the two circles, the more connection between the two cited references. Different colored nodes signify different meanings: the purple node represents early published articles, while the orange node represents recently published articles.

According to the citing frequency, a paper titled the Chicago Classification of Esophageal Motility Disorders, version 3.0, published in 2015, tops the list. The paper titled the Development of International Terminology and Definitions for Texture-Modified Foods and Thickened Fluids Used in Dysphagia Management: The IDDSI Framework ranks second. The top ten dysphagia-related articles accompanied by the most citations are shown in Table 6.

**Table 6:** The top ten cited references and centrality in the research field of dysphagia from 2012 to 2022.

**Ranking**	**Frequency**	**Centrality**	**Cited reference**	**Representative author (publication year)**
1	266	0.09	The Chicago classification of esophageal motility disorders, v3.0.	Kahrilas et al. (31)
2	187	0.02	Development of international terminology and definitions for texture-modified foods and thickened fluids used in dysphagia management: the IDDSI framework.	Cichero et al. (32)
3	155	0.03	Eosinophilic esophagitis: updated consensus recommendations for children and adults.	Liacouras et al. (33)
4	132	0.05	Chicago classification criteria of esophageal motility disorders defined in high resolution esophageal pressure topography.	Bredenoord et al. (34)
5	124	0.02	European society for swallowing disorders - European Union Geriatric Medicine Society white paper: oropharyngeal dysphagia as a geriatric syndrome.	Baijens et al. (35)
6	111	0.02	ACG clinical guideline: Evidenced based approach to the diagnosis and management of esophageal eosinophilia and eosinophilic esophagitis (EoE).	Dellon et al. (36)
7	108	0.02	The influence of food texture and liquid consistency modification on swallowing physiology and function: a systematic review.	Steele et al. (37)
8	87	0.02	Dysphagia: current reality and scope of the problem.	Clavé and Shaker (38)
9	79	0.01	Dysphagia in Parkinson's Disease.	Suttrup and Warnecke (39)
10	79	0.02	Post-stroke dysphagia: a review and design considerations for future trials.	Cohen et al. (40)

### Analysis of keywords

**Figure 9:**
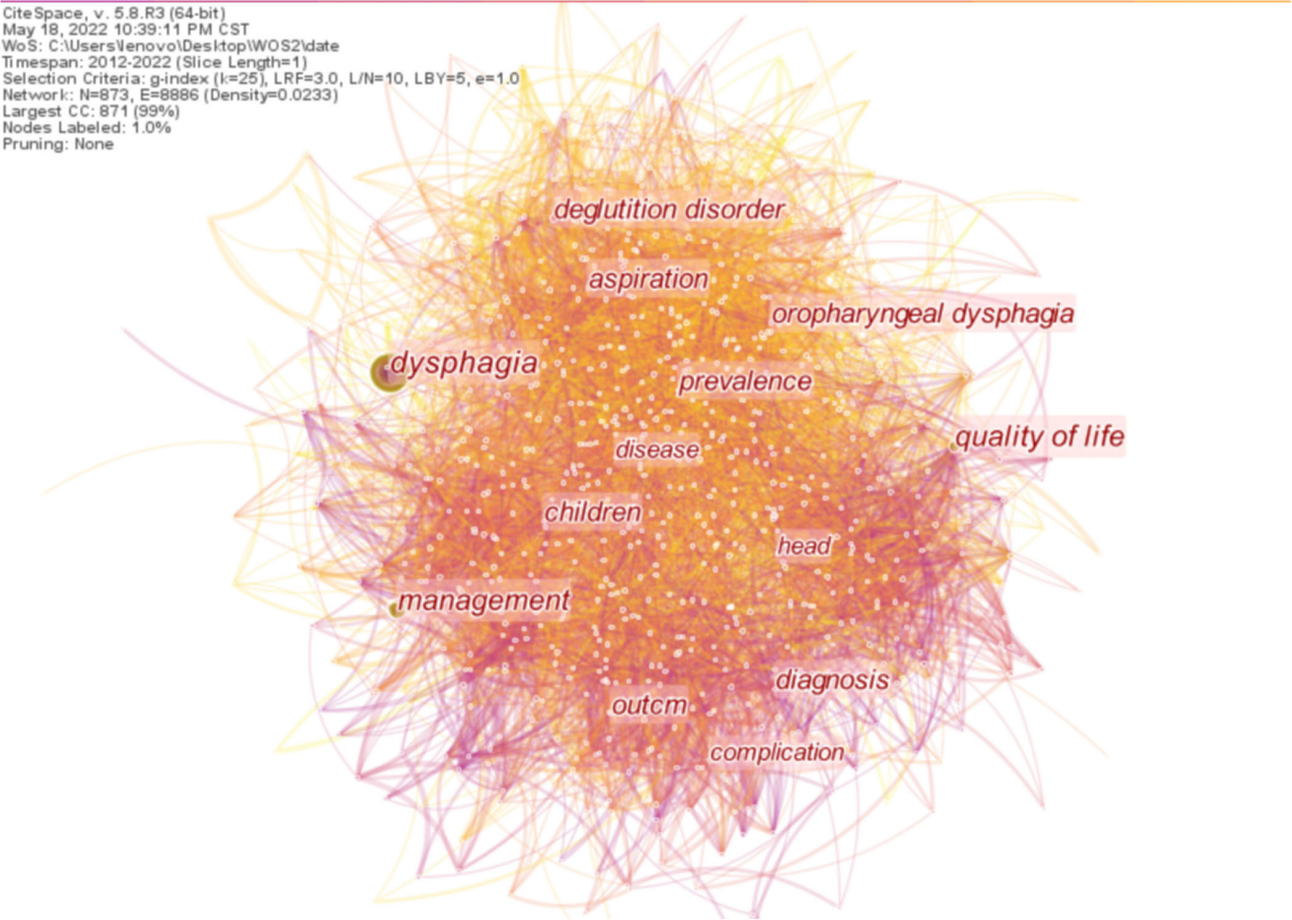
Map of keyword co-occurrence and cooperative relations on dysphagia from 2012 to 2022. The shorter the distance between the two circles, the more the cooperation between the two keywords.

By analyzing the frequency and centrality of keywords, the research frontier can be determined (25). Articles published from 2012 to 2022 were chosen with the time slice of 1 year, and the node type chose keywords for the analysis *via* CiteSpace. The resultant keyword co-occurrence map with a merged network of 873 nodes and 8,886 links is shown in Figure 9. We can observe that the most popular keywords are “dysphagia” with a frequency of 2,696, which is nearly two times that of ranking second on the list. These were followed by “management” and “quality of life.” Moreover, “deglutition disorder,” “diagnosis,” “aspiration,” and “prevalence” are other commonly used keywords.

**Figure 10:**
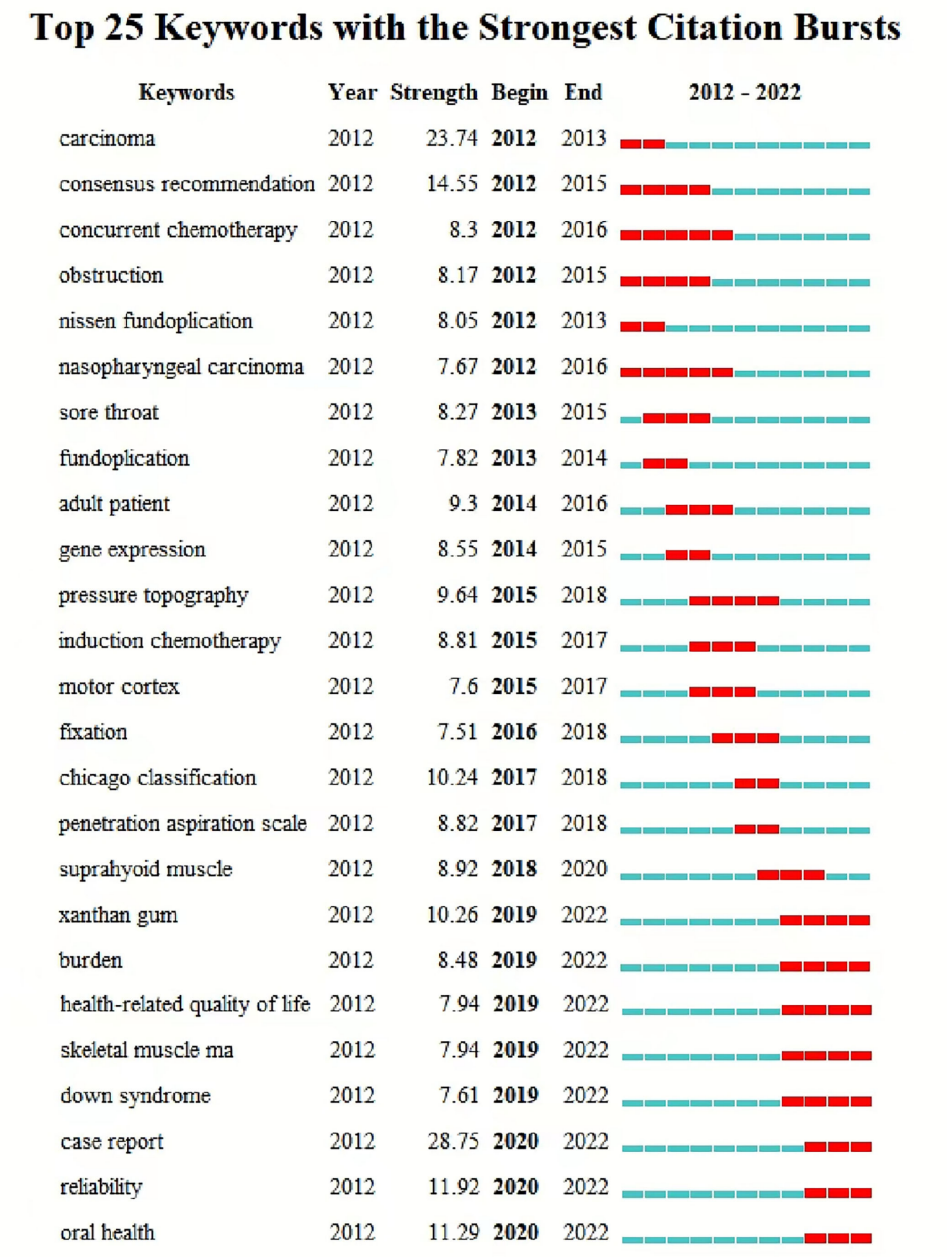
Top 25 keywords with the strongest citation bursts in published articles on dysphagia from 2012 to 2022. The timeline is depicted as a blue line, and the time interval that a subject was found to have a burst is shown as a red segment which indicated the beginning year, the ending year, and the duration of the burst.

The top ten keywords occurring in all the articles are shown in Table 7. “Burst” refers to the sudden increase in a period of time. Through keyword saliency detection, we can understand the development and changes of research hot spots, trends, and frontier dynamics within a certain period of time. The analysis of burst words helps detect the emergent words with high-frequency change rates and fast growth rates by investigating the time distribution of keywords and then analyzing the frontier fields and development trends of the discipline. The top 25 keywords with the strongest citation burst from 2012 to 2022 are shown in Figure 10. The blue bars refer to the time interval, while the red bars represent the duration of the citation burst. In the analysis of citation burst detection of the keywords, the case report is the strongest citation burst that appeared in 2012 with a burst strength of 28.75. It was followed by carcinoma with a burst strength of 23.74, consensus recommendation with a burst strength of 14.55, and reliability with a burst strength of 11.92. In addition to oral health, Chicago classification, pressure topography, and adult patient have an impact on related research.

**Table 7:** The top ten keywords and centrality in the research field of dysphagia from 2012 to 2022.

**Ranking**	**Keyword**	**Frequency**	**Centrality**
1	dysphagia	2,696	0.01
2	management	1,360	0.01
3	quality of life	1,217	0.01
4	deglutition disorder	1,028	0.01
5	diagnosis	899	0.01
6	aspiration	856	0.01
7	prevalence	830	0.01
8	children	807	0.01
9	outcome	760	0.01
10	oropharyngeal dysphagia	703	0.01

## Discussion

Dysphagia refers to an obstruction in the process of swallowing. It is a highly prevalent condition. Furthermore, the morbidity and mortality rates associated with dysphagia are high among the population, especially among the elderly (41). Recovering from neurological dysphagia is difficult (42). For the diagnosis of dysphagia, video fluoroscopy is traditionally used, and the modified barium swallowing study (MBSS) is generally the preferred tool. In recent years, fibroendoscopy has been increasingly used in clinical assessment. Regarding the treatment of swallowing disorders, in addition to swallowing training and nutrition intervention such as using texture-improved foods and thickened liquids, new rehabilitation approaches with stimulation technology also convey hope and will have a great significance on strategies in future treatment (43–45). Bibliometric analysis of dysphagia using CiteSpace from 2012 to 2021 was performed. We summarized the general information, research hot spots, and research trends.

We found that the number of articles published per year is increasing, particularly in the years between 2018 and 2021. This suggests that dysphagia is becoming more common and is being taken more seriously. It also leads to more active exploration and more in-depth insights that aid in understanding the swallowing difficulty. Given the current research trends, further research on dysphagia is likely to occur.

The analysis results *via* the CiteSpace project that the US leads the research in the field of dysphagia; a majority of the cited journals and institutions are from there. The most cited journal, Dysphagia, mainly publishes the latest cutting-edge original articles on the basic and clinical research of swallowing related to otorhinolaryngology, rehabilitation medicine, speech pathology, etc. It is the only journal dedicated to the study of multidisciplinary integration and is named after swallowing disorders. As the co-journal of the American Society for the Study of Swallowing Disorders, the European Society for the Study of Swallowing Disorders, and the Japanese Society for the Rehabilitation of Swallowing Disorders, it has established itself as a top academic journal in the field of swallowing disorders. Northwestern University is the most influential institution researching dysphagia, with further contributions from the Mayo Clinic, the University of Queensland, and Johns Hopkins University. The US and its institutions occupy a central position and maintain a high degree of cooperation with other countries and institutions. Collaboration helps researchers who investigated dysphagia share resources and exchange knowledge and ideas, which is crucial for further development in the field. Thus, stronger collaboration networks should be established among more countries, institutes, and authors.

Through the analysis of author nodes, the cooperative relationship between authors and others can be studied. Elizabeth Ward with 84 published articles and a centrality of 0.11 in the past 11 years is the most productive author in this field. Catriona Steele with 60 published articles and a centrality of 0.08 has also contributed significantly to the field. In terms of co-cited authors, Logemann JA, Rosenbek JC, and Kahrilas PJ have long-term cooperation with each other. Logemann JA specializes in the evaluation and treatment of dysphagia, Rosenbek JC has great insights into swallowing leakage and aspiration, and Kahrilas PJ specializes in esophageal dysphagia. They hold a high reputation in the field of swallowing disorders.

## Conclusion

This study conducted a comprehensive bibliometric analysis through CiteSpace and analyzed and created visualizations of 14,007 articles on dysphagia published from 2012 to 2022 that were retrieved from WOS Core Collections. We sought keyword cluster, publication year, country, institution, author, cited author, cite journal, and cited reference in the field. We probed into the trends and forefronts of swallowing disorders. As shown in Figure 1, over the recent 10 years, the number of publications on swallowing disorders has increased with each year, and the increase has been more rapid in recent years. The United States, Japan, and China were the leading countries in research on swallowing disorders. Dysphagia, Laryngoscope, Gastroenterology, and the New England Journal of Medicine emerged as the most influential journals in this field. The analysis results of keywords co-occurrence and citation burst keywords show that in recent years, much research has been conducted on the management, diagnosis, prevalence, outcome of dysphagia, quality of life, and deglutition disorder. Oral health, reliability, case report, and Down syndrome are emerging topics that deserve research focus. However, we acknowledge the limitations of this study resulting due to problems with software application and literature retrieval. CiteSpace analysis may be insufficient in some ways. For example, the software cannot clearly distinguish the first author from the corresponding author. When clustering, keywords can cause some overlaps between different categories of content. There are also several limitations to using CiteSpace: the learning curve is required to set accurate visualization parameters, and some maps and clusters are also complicated to interpret. Although we used the WoSCC for our bibliometric analysis, there are other public and commercially available bibliometric databases, like PubMed, Scopus, and Medline. We analyzed only “articles” studied in the WoSCC due to the huge number of literature. We believe that future research will be able to find effective solutions to overcome these problems, provide new ideas for conducting further research on swallowing disorders, and make more significant contributions to the development of swallowing.

In the analysis of cited references, high frequency and centrality reveal the research hot spots (46). For example, the Chicago Classification of Esophageal Motility Disorders, version 3.0 published in 2015 by Kahrilas PJ et al. (31) has been the gold standard in the field. However, there is ambiguity in the iterations of the Chicago classification; standardized and rigorous criteria for the patterns of disorders of peristalsis and obstruction at the EGJ need to improve. The article Development of International Terminology and Definitions for Texture-Modified Foods and Thickened Fluids Used in Dysphagia Management: the IDDSI Framework published in 2017 and written by Cichero JA et al. (32) put forward new ideas for dysphagia treatment.

Top-cited references and cited authors universally focused on the management and treatment of dysphagia. The analysis results of keyword co-occurrence and citation burst keywords show that in recent years, many studies have been conducted on the management, diagnosis, prevalence, outcome of dysphagia, quality of life, and deglutition disorder. Those hot keywords are certainly vital for the progress of dysphagia. However, emerging keywords such as impedance planimetry, rehabilitation nutrition, early management, functional outcomes, artificial intelligence, texture-modified food, and aged care, should also be paid attention to. Hence, we hope more studies will be conducted about them. Dysphagia is a painful illness that results in the loss of the pleasure of eating, a serious impact on the quality of life, and a serious financial burden on the family. Oral health, reliability, case report, and Down syndrome are rapidly emerging research topics.

To the best of our knowledge, currently, limited studies (47) exist on bibliometric analysis and visualization of dysphagia using CiteSpace, and this study is a meaningful attempt in that direction. This reveals the hot spots, trends, influential countries, institutions, and active authors in the field of dysphagia. However, we acknowledge the limitations faced by our study. We analyzed only the studies in WOS Core Collections with an article document type and time span from 2012 to 2022 because of software limitations. In addition, the literature retrieved may be missing or inconsistent with the subject due to issues related to the retrieval method. The results analyzed *via* CiteSpace may also be incomprehensive or incorrect. We will continue to explore, solve problems, and further promote the development of dysphagia.

## Data availability statement

The original contributions presented in the study are included in the article/supplementary material, further inquiries can be directed to the corresponding authors.

## Author contributions

WS and ZF designed the research subject. XK and GL conducted literature retrieval and screening. XD, YY, and SL provided guidance in statistical analysis. XK, NZ, GY, and GZ wrote the manuscript. LS, CZ, and WS critically revised the manuscript. All authors have read and approved the final manuscript.
